# A 47-year-old woman with dyspnea and bilateral lower extremity edema

**DOI:** 10.12701/jyms.2026.43.27

**Published:** 2026-03-29

**Authors:** Bogyeong Kim, Kang-Un Choi

**Affiliations:** 1 Yeungnam University College of Medicine, Daegu, Korea; 2 Department of Cardiology, Yeungnam University College of Medicine, Daegu, Korea

## Case presentation

A 47-year-old woman presented to the emergency department with progressively worsening bilateral lower extremity edema and dyspnea over the preceding 2 to 3 weeks. Her dyspnea worsened in the supine position and during ambulation. Lower extremity edema was pitting in nature. She had a history of anemia but had not received treatment. She reported adherence to a vegetarian diet for several decades.

**Fig. 1.**
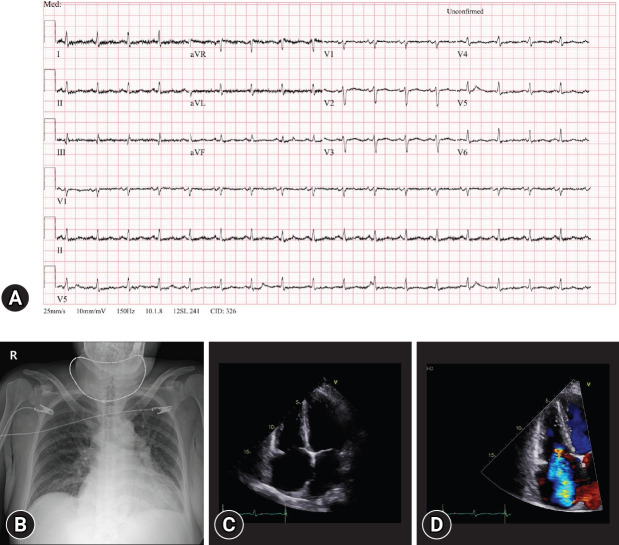
(A) Twelve-lead electrocardiogram demonstrating sinus tachycardia and right axis deviation. (B) Posteroanterior chest radiograph demonstrating significant cardiomegaly and increased pulmonary vasculature in both upper lung fields. These findings are highly suggestive of pulmonary congestion and interstitial edema. (C) Apical four-chamber echocardiographic view demonstrating biatrial enlargement with preserved left ventricular systolic function (left ventricular ejection fraction, 61%). (D) Color Doppler echocardiographic image demonstrating severe tricuspid regurgitation.

On presentation, her vital signs were as follows: blood pressure, 110/60 mmHg; heart rate, 106 beats/minute; body temperature, 37.2°C; and respiratory rate, 18 breaths/minute. Electrocardiography (ECG) revealed sinus tachycardia and right axis deviation (Fig. 1A). Chest radiography revealed cardiomegaly and increased pulmonary vasculature in both upper lung fields, suggesting pulmonary congestion and interstitial edema (Fig. 1B). Laboratory findings showed severe microcytic anemia with a hemoglobin level of 5.4 g/dL, hematocrit of 16.8%, mean corpuscular volume (MCV) of 73.2 fL, and mean corpuscular hemoglobin concentration of 32.9 g/dL. The N-terminal pro-B-type natriuretic peptide (NT-proBNP) level was markedly elevated at 2,850 pg/mL.

Initial transthoracic echocardiography revealed preserved left ventricle (LV) systolic function with a left ventricular ejection fraction (LVEF) of 61%. The transmitral E-wave velocity was 1.43 m/second, and the ratio of early diastolic mitral inflow velocity to early diastolic mitral annular velocity (E/e′ ratio), a surrogate marker of left atrial pressure, was 11.3. The left atrial volume index was increased to 51 mL/m², and right atrial enlargement was noted (Fig. 1C, Supplementary Video 1). The estimated pulmonary artery systolic pressure (PASP) was elevated to 67 mmHg. Severe tricuspid regurgitation (TR, Grade IV; Fig. 1D, Supplementary Video 2) and a small pericardial effusion were also present.

## Differential diagnosis

## 1. Iron deficiency anemia

Iron deficiency anemia (IDA) was strongly suspected because of severe microcytic anemia (hemoglobin, 5.4 g/dL; MCV, 73.2 fL). Iron assays, including assessments of total iron-binding capacity (TIBC), ferritin, and serum iron levels, were required to confirm iron depletion and identify the underlying cause.

## 2. Vitamin B12 deficiency anemia

Vitamin B12 deficiency causes megaloblastic anemia due to impaired DNA synthesis and may result from long-term vegetarianism or gastrointestinal malabsorption. Research indicates that approximately 52% of vegans and 7% of vegetarians are vitamin B12 deficient (serum vitamin B12 <118 pmol/L) [1]. Given the patient’s long-standing strict vegetarian diet, vitamin B12 deficiency was considered.

## 3. Heart failure with preserved ejection fraction

Heart failure (HF) is a clinical syndrome characterized by the inability of the heart to pump sufficient blood to meet metabolic demands. It typically presents as dyspnea, orthopnea, peripheral edema, and elevated natriuretic peptide levels [2]. The patient experienced worsening dyspnea in the supine position and bilateral pitting edema. Cardiomegaly and increased pulmonary vasculature, suggestive of pulmonary congestion, were noted on chest radiography (Fig. 1B), and NT-proBNP levels were markedly elevated to 2,850 pg/mL, strongly suggesting HF. The HF subtype is determined by LVEF on echocardiography. In this case, the LVEF was 61%, supporting a diagnosis of HF with preserved ejection fraction (HFpEF).

## 4. Pulmonary hypertension

Pulmonary hypertension (PH) was also considered because the ECG showed right axis deviation, and echocardiography demonstrated right atrial enlargement, elevated PASP, and severe TR. Given the evidence of increased left atrial pressure, Group 2 PH due to left heart disease was considered.

## Diagnosis

The patient reported a history of chronic menorrhagia, and computed tomography was performed to investigate the underlying cause, which revealed a mass suspected of being a uterine leiomyoma (Supplementary Fig. 1). A gynecological consultation was subsequently performed, which confirmed that the uterine leiomyoma was the definitive source of chronic bleeding, leading to severe IDA. Vitamin B12 (343 pg/mL) and folate (12 ng/mL) levels were normal, whereas TIBC was elevated to 532 μg/dL, ferritin was 5.19 ng/mL, and serum iron was decreased to 13 μg/dL. HF was suspected based on the presence of dyspnea, leg edema, and elevated NT-proBNP levels. Echocardiography showed preserved LVEF (61%), right ventricle (RV) dilatation, and elevated PASP, supporting the diagnosis of high-output HF secondary to chronic IDA.

## Treatment and prognosis

Management of high-output HF is primarily directed at identifying and treating the underlying etiology, with supportive measures to control volume status [3]. High-output HF induced by anemia typically improves once the primary hematological deficit is resolved. However, in cases of symptomatic high-output syndrome, as was observed in this patient, early blood transfusion may be required. Because this patient already had volume overload, transfusion was administered cautiously at a slow rate with concurrent diuretics, as needed, to avoid transfusion-associated circulatory overload while restoring oxygen-carrying capacity. During the initial phase of hospitalization, packed red blood cell transfusions were cautiously administered to correct severe anemia, followed by intravenous iron therapy to facilitate definitive hematological recovery. To manage systemic congestion, loop diuretics were prioritized. Subsequently, a mineralocorticoid receptor antagonist and an angiotensin receptor-neprilysin inhibitor (ARNI) were added according to the patient’s clinical course.

Follow-up echocardiography on the seventh day of hospitalization demonstrated normal LV systolic function, with an LVEF of 58%. The transmitral E-wave velocity decreased to 1.09 m/second, and the E/e ratio remained borderline at 14.0. The left atrial volume index decreased to 41 mL/m². Although the right atrial size was reduced compared to the baseline value, it remained dilated. The PASP improved to 52 mmHg, and TR was downgraded to Grade II. The previously noted small pericardial effusion had resolved. On the same day, hemoglobin was found to have increased to 11.6 g/dL, and MCV had normalized to 85.6 fL. Clinically, the patient’s dyspnea and peripheral edema had resolved. After confirming hemodynamic stability, the patient was discharged with plans for regular outpatient follow-up to monitor for the recurrence of anemia and further improvements in right-sided heart pressure and TR. At the 1-year follow-up, the patient’s NT-proBNP level had decreased to 200 pg/mL, and echocardiography demonstrated an improved E/e′ ratio of 11.6.

## Discussion

HFpEF is a heterogeneous clinical syndrome characterized by symptoms of HF despite a preserved LVEF [4]. It is increasingly understood as a syndrome driven by multiple comorbidities rather than a single disease. Its key pathophysiology involves diastolic dysfunction and reduced LV compliance, resulting in elevated filling pressures [5]. This increases the left atrial and pulmonary venous pressures, causing dyspnea and exercise intolerance. Therefore, the diagnosis of HFpEF should be based on clinical symptoms, elevated NT-proBNP levels [2, 6], echocardiographic evidence of increased filling pressures, PH [6], and not LVEF alone.

**Fig. 2.**
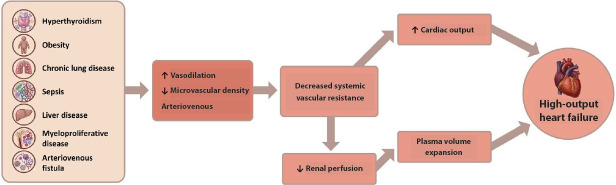
Pathophysiologic mechanisms of high-output heart failure. Various conditions such as hyperthyroidism, obesity, liver disease, chronic lung disease, sepsis, myeloproliferative disorders, and arteriovenous fistulas can lead to systemic vasodilation and decreased systemic vascular resistance. This results in increased cardiac output, renal hypoperfusion, plasma volume expansion, and ultimately high-output heart failure.

In the present case, severe IDA was identified as a critical comorbidity. IDA is the most common cause of microcytic anemia in adults, and chronic menorrhagia is a common etiology in women of childbearing age [7]. In this patient, chronic menorrhagia due to a uterine leiomyoma led to severe IDA. Although chronic anemia may be compensated for when it develops gradually, a reduction in hemoglobin to approximately 6 g/dL can induce a high-output state and precipitate HF. High-output HF is characterized by increased cardiac output and reduced systemic vascular resistance. Severe anemia reduces the oxygen-carrying capacity of blood, leading to tissue hypoxia. As a compensatory response, this triggers peripheral vasodilation and subsequently decreases systemic vascular resistance [3,7]. This persistent high-output state triggers neurohormonal activation, specifically involving the renin-angiotensin-aldosterone system and antidiuretic hormones, leading to excessive sodium and water retention. This ultimately results in hypervolemia, elevated filling pressures, and congestion [8]. High-output HF has diverse causes, including anemia, obesity, liver disease, chronic lung disease, sepsis, beriberi, hyperthyroidism, myeloproliferative disorders, arteriovenous fistulas, and Paget’s disease of bone (Fig. 2) [8]. These conditions increase cardiac demand or reduce systemic vascular resistance.

Management of high-output HF requires correction of the underlying cause and relief of congestion [3]. Therapeutic strategies should extend beyond symptom relief with diuretics to address the primary drivers of the high-output state. Notably, the 2023 European Society of Cardiology focused update also emphasizes iron deficiency management in HF and recommends iron supplementation to improve symptoms and outcomes [9]. ARNI use in this patient was consistent with current HFpEF management. Clinical trials, such as PARAGON-HF, have shown that ARNI therapy may reduce HF hospitalization, especially in women and patients with LVEF near the lower end of the preserved range [10]. In this patient, anemia was corrected through transfusion and iron supplementation, while diuretics were concurrently administered to manage volume overload. The subsequent reduction in PASP, improvement in TR, and resolution of clinical symptoms suggest that the correction of anemia contributed significantly to the improved underlying HF pathophysiology.

In conclusion, this case underscores the importance of systematically evaluating comorbidities and reversible risk factors in patients with HF and normal LVEF, rather than classifying the pathophysiology based on LVEF values alone. Severe IDA should be regarded not merely as a coexisting condition but also as a significant contributor to the HF phenotype through the induction of a high-output state. Therefore, a personalized approach to assess and manage correctable risk factors is essential for the clinical management of HFpEF.

## Educational pearls

### 1. Severe iron deficiency anemia can present as high-output heart failure with preserved ejection fraction

Profound chronic anemia reduces oxygen-carrying capacity, resulting in compensatory increases in cardiac output and decreased systemic vascular resistance. Persistent high-output physiology may lead to PH, RV dilation, and systemic congestion, despite preserved LV systolic function.

### 2. Elevated N-terminal pro-B-type natriuretic peptide with normal left ventricular ejection fraction does not exclude clinically significant heart failure

Patients presenting with dyspnea, edema, and elevated natriuretic peptide levels may have HF despite a normal LVEF. Careful assessment of volume status, filling pressures, pulmonary pressures, and potential secondary causes (e.g., anemia and thyroid disease) is essential.

### 3. Treatment of high-output heart failure requires correction of the underlying cause

Although diuretics alleviate the symptoms of congestion, definitive management depends on addressing the precipitating condition. In anemia-induced high-output HF, blood transfusion and iron replacement can reverse hemodynamic abnormalities and improve PH and TR.

## Question 1

A 47-year-old woman with a history of several years of heavy menstrual bleeding presented with progressive dyspnea and bilateral leg edema. Laboratory tests revealed a hemoglobin level of 5.4 g/dL and an NT-proBNP level of 2,850 pg/mL. Echocardiography revealed preserved LVEF (61%), RV enlargement, and elevated pulmonary artery pressure.Which of the following is the most likely underlying cause of her HF?

- A. Severe chronic IDA
- B. Acute myocardial infarction
- C. Primary dilated cardiomyopathy
- D. Hypertrophic cardiomyopathy
- E. Acute viral myocarditis

A

## Question 2

Which of the following statements regarding NT-proBNP is the most accurate?

- A. It is elevated only in patients with reduced ejection fraction
- B. It is specific for ischemic heart disease
- C. It is unaffected by volume status
- D. It can be elevated in HFpEF
- E. It is produced primarily by the atria

D

## Supplementary materials

Supplementary Fig. 1 and Supplementary Videos 1 and 2 can be found at https://doi.org/10.12701/jyms.2026.43.27.

Supplementary Fig 1. Abdominopelvic computed tomography.

Supplementary Video 1. Apical four-chamber echocardiographic view.

Supplementary Video 2. Color Doppler echocardiography demonstrating severe tricuspid regurgitation.
