# Supplementary material for: A 47-year-old woman with dyspnea and bilateral lower extremity edema
Source: J Yeungnam Med Sci. 2026 Mar 29;43:27. doi: 10.12701/jyms.2026.43.27 (PMC13399056; doi:10.12701/jyms.2026.43.27)
Supplement: Supplementary Fig 1. — Abdominopelvic computed tomography. [file jyms-2026-43-27-Supplementary-Fig-1.pdf]

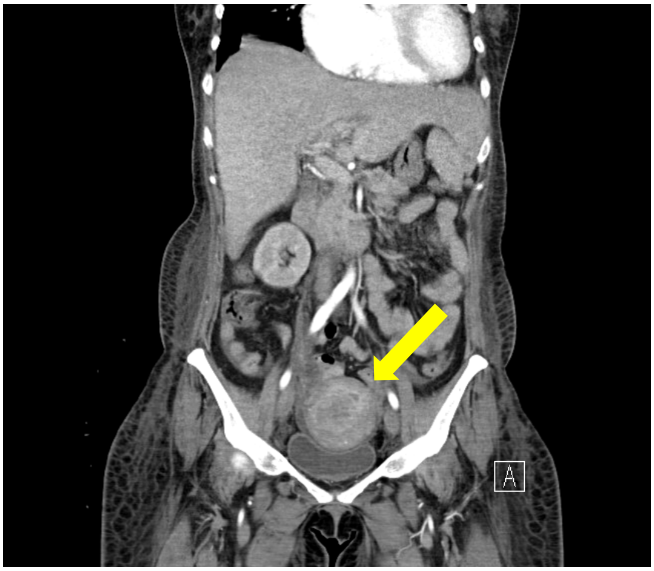

Supplementary Fig. 1. Abdominopelvic computed tomography. Coronal contrast-enhanced computed tomography scan demonstrating the abdominal and pelvic regions, shows a uterine mass consistent with a leiomyoma (arrow).
